# Improving the quality of protein structure models by selecting from alignment alternatives

**DOI:** 10.1186/1471-2105-7-364

**Published:** 2006-07-27

**Authors:** Ingolf Sommer, Stefano Toppo, Oliver Sander, Thomas Lengauer, Silvio CE Tosatto

**Affiliations:** 1 Department of Computational Biology and Applied Algorithmics, Max-Planck-lnstitute for Informatics, Stuhlsatzenhausweg 85, D-66123 Saarbrücken, Germany; 2 Department of Biological Chemistry, University of Padova, via U. Bassi 58/b, 1-35121 Padova, Italy; 3 Department of Biology and CRIBI Biotechnology Centre University of Padova, V.le G. Colombo 3, I-35131 Padova, Italy

## Abstract

**Background:**

In the area of protein structure prediction, recently a lot of effort has gone into the development of Model Quality Assessment Programs (MQAPs). MQAPs distinguish high quality protein structure models from inferior models. Here, we propose a new method to use an MQAP to improve the quality of models. With a given target sequence and template structure, we construct a number of different alignments and corresponding models for the sequence. The quality of these models is scored with an MQAP and used to choose the most promising model. An SVM-based selection scheme is suggested for combining MQAP partial potentials, in order to optimize for improved model selection.

**Results:**

The approach has been tested on a representative set of proteins. The ability of the method to improve models was validated by comparing the MQAP-selected structures to the native structures with the model quality evaluation program *TM*-score. Using the SVM-based model selection, a significant increase in model quality is obtained (as shown with a Wilcoxon signed rank test yielding p-values below 10^-15^). The average increase in *TM*score is 0.016, the maximum observed increase in *TM*-score is 0.29.

**Conclusion:**

In template-based protein structure prediction alignment is known to be a bottleneck limiting the overall model quality. Here we show that a combination of systematic alignment variation and modern model scoring functions can significantly improve the quality of alignment-based models.

## 1 Background

Protein structure prediction by comparative modeling and/or fold recognition consists of three largely independent steps: (1) Postulating the structural similarity of the target protein sequence with a known template structure on the basis of a significant alignment score between the two protein sequences. (2) This or a different alignment serves as a basis for model construction. In this process residues in the target sequence that are aligned to residues in the template structure are mapped on the corresponding coordinates in the structure. (3) Finally, unmapped regions are filled in, breaks in the backbone are mended, and the overall model is refined.

Thus the quality of the alignment in the second step has an essential impact on the quality of the resulting model. The continual benchmarks in the biannual CASP assessment of protein structure prediction methods witness that there is significant progress in identifying suitable templates [1], due in part to the introduction of profile-profile alignment methods [2-5] and the sophisticated construction of profiles [6]. While CASP assessors found little improvement in the predicted models [7], they found steady progress in alignment quality over the years [8].

The optimal alignment resulting from an algorithm with a specific optimized parameter setting is not always the best choice for model creation. Jaroszewski et al. have set up a computational experiment in which they sample a huge conformational space (size up to 10^10^) of alternative alignments by combining an approach of varying parameters (such as gap penalties and substitution matrices) with an iterative approach of penalizing previously visited regions of the sample space [9]. The study states that there exist alignments surpassing the original alignments in quality for about 50% of the protein pairs. Contreras-Moreira and coworkers [10] as well as John and Sali [11] propose genetic algorithms for constructing a large number of alternative alignments by recombining an initial set of alignments. A common problem of these approaches is the selection of the alignment allowing for the construction of the final model.

Recently, a lot of effort has gone into the development of Model Quality Assessment Programs (MQAPs) [12-14]. MQAPs are computer programs that receive as input a 3D model of a protein structure and produce as output a real number representing the quality of the model [15]. We will refer to this number as the model score. In contrast to model evaluation programs, like GDT [16], MaxSub [17], or TM-score [18], which assess the quality of the model by comparing it to the native structure, MQAPs do not compare to the native structure. Instead, they estimate the quality of a proposed model without knowledge of the native structure. Unlike scoring functions in sequence-to-structure alignment and to physical energy functions, MQAPs operate on an intermediate level – they are more flexible than a sequence-to-structure alignment function as the dynamic programming paradigm used in alignment computation imposes the requirement of prefix optimality which is not required in MQAPs. MQAPs aim at scoring the quality of predicted models. Typically, MQAPs use one or more different statistical potentials, representing information coded in protein structures [19,20,12,13]. Different MQAPs were recently tested in CAFASP-4 as meta-selectors for pinpointing high quality models from the ensemble of models proposed by different automated servers [15,13,21] proving that MQAPs are highly effective selectors.

## 2 Results

### 2.1 Overview of protocol and evaluation

In this manuscript we propose and validate a protocol for improving alignments in step (2) of comparative modeling or fold recognition. Optimization is achieved by generating alternative alignment-based models for a target sequence and selecting the most promising model using an MQAP.

Ensembles of alternative alignments are generated with the state-of-the-art profile-profile alignment method Arby [22,23] by varying parameters. Apart from the Arby default, we suggest two different procedures for generating alternative alignments: *PVS *varies the parameters in the profile-profile alignment method slightly, whereas *PVH *varies the parameters heavily. Each procedure reports an ensemble of distinct alignments. For each alignment a model is constructed (see Methods for details, as well as Table 1 for an overview of the parameters used in *PVS *and *PVH*).

The ensembles of alternative models typically contain models with higher quality as well as models with lower quality than the standard Arby model. The FRST [13] MQAP program is applied to scoring the quality of the models. By choosing the model with the best model score according to the FRST potential, we can select a promising model for each target. These selected models are potentially improved with respect to the Arby default model. Additionally, we developed an SVM-based selection mechanism. A support vector machine (SVM) is trained on the model scores and on the FRST partial potentials for recognizing the models with increased quality.

The performance of the protocol is evaluated by comparing the chosen models to the previously withheld native structures. The comparison is performed with the model evaluation program *TM*-score [18], its score reflecting the "real" quality of the models. The *TM*-score always lies in the interval (0,1], where the upper limit stands for a model perfectly superposable with the structure. This allows for comparing the quality of the generated and selected models with the quality of the default Arby models and for assessing the significance of the selection process.

The protocol was evaluated on a set of 1612 target sequences with known structures (see Methods). For each target *t *we computed the Arby default model *d*(*t*) and exercised the two model generation procedures *PVS *and *PVH *resulting in two ensembles of models E_*PVS*_(*t*) and E_*PVH*_(*t*) per target. Summary statistics of the number of models per target are given in Table 2.

### 2.2 Evaluation of model generation: quality of generated models

First, we analyze the quality of the model generation procedures. The key ideas are to count per target the number of models with increased quality, and to measure the average difference of model quality with respect to the default model in terms of *TM*-score.

#### 2.2.1 Analysis per target

For a target *t*, we denote the quality of a model *ml *by *TM*(*ml*), where greater *TM*-score is better. The relative frequency of models per target with a quality measure above the Arby default is defined as

fptE,>(t)=1|E(t)|∑ml∈E(t)[TM(ml)>TM(d(t))],
 MathType@MTEF@5@5@+=feaafiart1ev1aaatCvAUfKttLearuWrP9MDH5MBPbIqV92AaeXatLxBI9gBaebbnrfifHhDYfgasaacH8akY=wiFfYdH8Gipec8Eeeu0xXdbba9frFj0=OqFfea0dXdd9vqai=hGuQ8kuc9pgc9s8qqaq=dirpe0xb9q8qiLsFr0=vr0=vr0dc8meaabaqaciaacaGaaeqabaqabeGadaaakeaacqWGMbGzcqWGWbaCcqWG0baDdaWgaaWcbaGaemyrauKaeiilaWIaeyOpa4dabeaakiabcIcaOiabdsha0jabcMcaPiabg2da9maalaaabaGaeGymaedabaGaeiiFaWNaemyrauKaeiikaGIaemiDaqNaeiykaKIaeiiFaWhaamaaqafabaGaei4waSLaemivaqLaemyta0KaeiikaGIaemyBa0MaemiBaWMaeiykaKIaeyOpa4JaemivaqLaemyta0KaeiikaGIaemizaqMaeiikaGIaemiDaqNaeiykaKIaeiykaKIaeiyxa0LaeiilaWcaleaacqWGTbqBcqWGSbaBcqGHiiIZcqWGfbqrcqGGOaakcqWG0baDcqGGPaqkaeqaniabggHiLdaaaa@5EB8@

where *d*(*t*) is the default Arby model, *E*(*t*) is an ensemble of models for the target, and [*x*] is the Iverson bracket defined for arbitrary propositions *x *as

[x]={1if x is true0else.
 MathType@MTEF@5@5@+=feaafiart1ev1aaatCvAUfKttLearuWrP9MDH5MBPbIqV92AaeXatLxBI9gBaebbnrfifHhDYfgasaacH8akY=wiFfYdH8Gipec8Eeeu0xXdbba9frFj0=OqFfea0dXdd9vqai=hGuQ8kuc9pgc9s8qqaq=dirpe0xb9q8qiLsFr0=vr0=vr0dc8meaabaqaciaacaGaaeqabaqabeGadaaakeaacqGGBbWwcqWG4baEcqGGDbqxcqGH9aqpdaGabaqaauaabmqaciaaaeaacqaIXaqmaeaacqqGPbqAcqqGMbGzcqqGGaaicqWG4baEcqqGGaaicqqGPbqAcqqGZbWCcqqGGaaicqqG0baDcqqGYbGCcqqG1bqDcqqGLbqzaeaacqaIWaamaeaacqqGLbqzcqqGSbaBcqqGZbWCcqqGLbqzaaGaeiOla4cacaGL7baaaaa@49E3@

Similarly, we consider the relative frequency *fpt*_*E*,<_(*t*) of models with a quality below that of the Arby default models.

The average within an ensemble *E*(*t*) of quality improvement of a model over the default Arby model is

qirE(t)=1|E(t)|∑ml∈E(t)(TM(ml)−TM(d(t))).
 MathType@MTEF@5@5@+=feaafiart1ev1aaatCvAUfKttLearuWrP9MDH5MBPbIqV92AaeXatLxBI9gBaebbnrfifHhDYfgasaacH8akY=wiFfYdH8Gipec8Eeeu0xXdbba9frFj0=OqFfea0dXdd9vqai=hGuQ8kuc9pgc9s8qqaq=dirpe0xb9q8qiLsFr0=vr0=vr0dc8meaabaqaciaacaGaaeqabaqabeGadaaakeaacqWGXbqCcqWGPbqAcqWGYbGCdaWgaaWcbaGaemyraueabeaakiabcIcaOiabdsha0jabcMcaPiabg2da9maalaaabaGaeGymaedabaGaeiiFaWNaemyrauKaeiikaGIaemiDaqNaeiykaKIaeiiFaWhaamaaqafabaGaeiikaGIaemivaqLaemyta0KaeiikaGIaemyBa0MaemiBaWMaeiykaKIaeyOeI0IaemivaqLaemyta0KaeiikaGIaemizaqMaeiikaGIaemiDaqNaeiykaKIaeiykaKIaeiykaKcaleaacqWGTbqBcqWGSbaBcqGHiiIZcqWGfbqrcqGGOaakcqWG0baDcqGGPaqkaeqaniabggHiLdGccqGGUaGlaaa@5BF9@

We define an indicator function whether a better model for a target *t *exists in the ensemble *E*(*t*).

*fb*_*E*_(*t*) = [∃*ml *∈ *E*(*t*) : *TM*(*ml*) > *TM*(*d*(*t*))]

and compute the quality improvement that is theoretically possible

qibE(t)=max⁡ml∈E(t)TM(ml)−TM(d(t)).
 MathType@MTEF@5@5@+=feaafiart1ev1aaatCvAUfKttLearuWrP9MDH5MBPbIqV92AaeXatLxBI9gBaebbnrfifHhDYfgasaacH8akY=wiFfYdH8Gipec8Eeeu0xXdbba9frFj0=OqFfea0dXdd9vqai=hGuQ8kuc9pgc9s8qqaq=dirpe0xb9q8qiLsFr0=vr0=vr0dc8meaabaqaciaacaGaaeqabaqabeGadaaakeaacqWGXbqCcqWGPbqAcqWGIbGydaWgaaWcbaGaemyraueabeaakiabcIcaOiabdsha0jabcMcaPiabg2da9uaabmqabiaaaeaadaWfqaqaaiGbc2gaTjabcggaHjabcIha4bWcbaGaemyBa0MaemiBaWMaeyicI4SaemyrauKaeiikaGIaemiDaqNaeiykaKcabeaaaOqaaiabdsfaujabd2eanjabcIcaOiabd2gaTjabdYgaSjabcMcaPiabgkHiTiabdsfaujabd2eanjabcIcaOiabdsgaKjabcIcaOiabdsha0jabcMcaPiabcMcaPaaacqGGUaGlaaa@543C@

#### 2.2.2 Performance over all targets

The frequency *fpt *was defined per target and its average over all targets is fpt¯=1n∑fpt
 MathType@MTEF@5@5@+=feaafiart1ev1aaatCvAUfKttLearuWrP9MDH5MBPbIqV92AaeXatLxBI9gBaebbnrfifHhDYfgasaacH8akY=wiFfYdH8Gipec8Eeeu0xXdbba9frFj0=OqFfea0dXdd9vqai=hGuQ8kuc9pgc9s8qqaq=dirpe0xb9q8qiLsFr0=vr0=vr0dc8meaabaqaciaacaGaaeqabaqabeGadaaakeaadaqdaaqaaiabdAgaMjabdchaWjabdsha0baacqGH9aqpdaWcaaqaaiabigdaXaqaaiabd6gaUbaadaaeabqaaiabdAgaMjabdchaWjabdsha0bWcbeqab0GaeyyeIuoaaaa@3A9A@. While *fpt *describes the frequency of better models per target, fb¯=1n∑fb
 MathType@MTEF@5@5@+=feaafiart1ev1aaatCvAUfKttLearuWrP9MDH5MBPbIqV92AaeXatLxBI9gBaebbnrfifHhDYfgasaacH8akY=wiFfYdH8Gipec8Eeeu0xXdbba9frFj0=OqFfea0dXdd9vqai=hGuQ8kuc9pgc9s8qqaq=dirpe0xb9q8qiLsFr0=vr0=vr0dc8meaabaqaciaacaGaaeqabaqabeGadaaakeaadaqdaaqaaiabdAgaMjabdkgaIbaacqGH9aqpdaWcaaqaaiabigdaXaqaaiabd6gaUbaadaaeabqaaiabdAgaMjabdkgaIbWcbeqab0GaeyyeIuoaaaa@3780@ reflects the fraction of targets that have a model with a quality above the Arby default within the ensemble of constructed models.

When selecting models randomly, an average quality improvement of qir¯=1n∑qir
 MathType@MTEF@5@5@+=feaafiart1ev1aaatCvAUfKttLearuWrP9MDH5MBPbIqV92AaeXatLxBI9gBaebbnrfifHhDYfgasaacH8akY=wiFfYdH8Gipec8Eeeu0xXdbba9frFj0=OqFfea0dXdd9vqai=hGuQ8kuc9pgc9s8qqaq=dirpe0xb9q8qiLsFr0=vr0=vr0dc8meaabaqaciaacaGaaeqabaqabeGadaaakeaadaqdaaqaaiabdghaXjabdMgaPjabdkhaYbaacqGH9aqpdaWcaaqaaiabigdaXaqaaiabd6gaUbaadaaeabqaaiabdghaXjabdMgaPjabdkhaYbWcbeqab0GaeyyeIuoaaaa@3AA2@ is obtained. When selecting models optimally, an average quality improvement of qib¯=1n∑qib
 MathType@MTEF@5@5@+=feaafiart1ev1aaatCvAUfKttLearuWrP9MDH5MBPbIqV92AaeXatLxBI9gBaebbnrfifHhDYfgasaacH8akY=wiFfYdH8Gipec8Eeeu0xXdbba9frFj0=OqFfea0dXdd9vqai=hGuQ8kuc9pgc9s8qqaq=dirpe0xb9q8qiLsFr0=vr0=vr0dc8meaabaqaciaacaGaaeqabaqabeGadaaakeaadaqdaaqaaiabdghaXjabdMgaPjabdkgaIbaacqGH9aqpdaWcaaqaaiabigdaXaqaaiabd6gaUbaadaaeabqaaiabdghaXjabdMgaPjabdkgaIbWcbeqab0GaeyyeIuoaaaa@3A62@ is obtained, imposing a theoretical upper bound to what is feasible with MQAP selection on the alignments generated as proposed. For the two procedures *PVS *and *PVH *generating alignment-based models these numbers are listed in Table 3.

**Figure 1:**
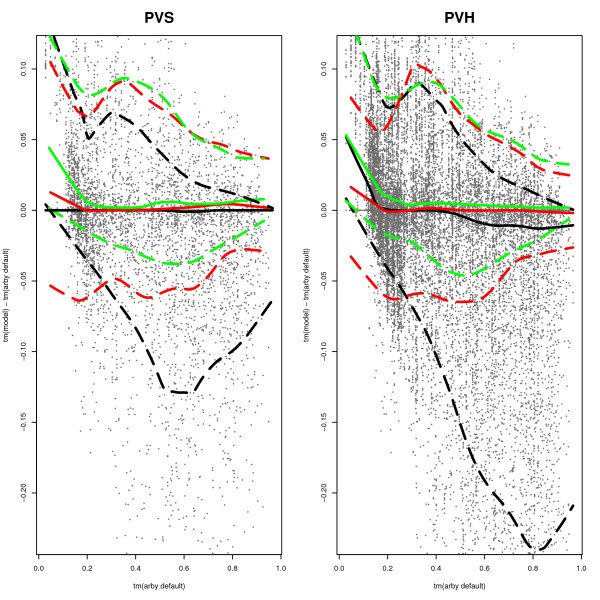
Overview of model quality improvement with respect to the model difficulty. Left for PVS, right for PVH analogously. Each dot corresponds to a model where the x-coordinate is the *TM*-score of the corresponding default Arby model and the y-coordinate is the *TM*-score improvement with respect to this default model. Smoothed quantile lines are shown for the 10% (lower dashed), 50% (middle), 90% (upper dashed) quantiles of the models within a sliding window of size 0.15. Black lines represent all models, red lines represent the models selected using FRST, green lines represent the models selected using the SVM approach. For the smoothing evaluations are made at 1000 equidistant points and the resulting quantiles are smoothed with a lowess function (local linear scatter plot smoother). **Interpretation: **The *TM*-score of the Arby default gives an indication of how difficult it is to find the right template for a target. For the selection methods random, FRST, and SVM, this plot shows the potential improvement with respect to difficulty of the target. For PVH, more models are generated below default. For both PVS and PVH, the SVM selection performs better than FRST selection, and FRST performs better than random.

In order to visualize the distributions of model quality, in Figure 1 for each target *t *the *TM*-score of the default Arby model *s *plotted versus the *TM*-score improvements of the models constructed for that target. The scatter plots in Figure 1 along with Table 3 clearly indicate that better models are generated for a large fraction of the targets. Summing up, the above-mentioned procedures generate models with a better quality than the Arby default models, but identification of the improved models among the generated models is a hard task as analyzed in the next section.

### 2.3 Evaluation of model selection

In the following, we analyze how well the model selection procedure works on the models generated with procedures *PVS *and *PVH*. The key ideas are to count for how many targets an improved model is selected, and to measure the quality improvement with respect to the default model in terms of *TM*-score. We perform the analysis for the selection based on the FRST potential and then repeat it analogously for the SVM based selection.

#### 2.3.1 Analysis per target

Identification of the best model per target can be performed based on the FRST MQAP scores. For each target *t*, we select the model *s *with the lowest estimated *frst *energy

sE,frst(t)=arg⁡min⁡ml∈E(t)frst(ml),
 MathType@MTEF@5@5@+=feaafiart1ev1aaatCvAUfKttLearuWrP9MDH5MBPbIqV92AaeXatLxBI9gBaebbnrfifHhDYfgasaacH8akY=wiFfYdH8Gipec8Eeeu0xXdbba9frFj0=OqFfea0dXdd9vqai=hGuQ8kuc9pgc9s8qqaq=dirpe0xb9q8qiLsFr0=vr0=vr0dc8meaabaqaciaacaGaaeqabaqabeGadaaakeaacqWGZbWCdaWgaaWcbaGaemyrauKaeiilaWIaemOzayMaemOCaiNaem4CamNaemiDaqhabeaakiabcIcaOiabdsha0jabcMcaPiabg2da9uaabmqabiaaaeaadaWfqaqaaiGbcggaHjabckhaYjabcEgaNjGbc2gaTjabcMgaPjabc6gaUbWcbaGaemyBa0MaemiBaWMaeyicI4SaemyrauKaeiikaGIaemiDaqNaeiykaKcabeaaaOqaaiabdAgaMjabdkhaYjabdohaZjabdsha0jabcIcaOiabd2gaTjabdYgaSjabcMcaPaaacqGGSaalaaa@5607@

since lower *frst *is better. In the supplementary material (see additional file supplement) we analyze the FRST partial potentials in more detail.

In order to count the number of occurrences in which this is an improvement of model quality measured in *TM*-score, we define the indicator functions *fim *as follows:

*fim*_*E,frst*,>_(*t*) = [*TM*(*s*_*E,frst*_(*t*)) > *TM*(*d*(*t*))]

*fim*_*E,frst*,=_(*t*) = [*TM*(*s*_*E,frst*_(*t*)) = *TM*(*d*(*t*))]

*fim*_*E,frst*,<_(*t*) = [*TM*(*s*_*E,frst*_(*t*)) <*TM*(*d*(*t*))]

These functions indicate whether the model selected by the MQAP is of higher, equal, or lower quality than the Arby default.

While *fim *serves to count the number of targets which improve, we use the measure *qim *to quantify the improvement of model quality with respect to the default Arby model:

*qim*_*E,frst*_(*t*) = *TM*(*s*_*E,frst*_(*t*)) - *TM*(*d*(*t*)).

#### 2.3.2 Performance over all targets

Across all targets, fim¯>=1n∑fim>
 MathType@MTEF@5@5@+=feaafiart1ev1aaatCvAUfKttLearuWrP9MDH5MBPbIqV92AaeXatLxBI9gBaebbnrfifHhDYfgasaacH8akY=wiFfYdH8Gipec8Eeeu0xXdbba9frFj0=OqFfea0dXdd9vqai=hGuQ8kuc9pgc9s8qqaq=dirpe0xb9q8qiLsFr0=vr0=vr0dc8meaabaqaciaacaGaaeqabaqabeGadaaakeaadaqdaaqaaiabdAgaMjabdMgaPjabd2gaTbaadaWgaaWcbaGaeyOpa4dabeaakiabg2da9maalaaabaGaeGymaedabaGaemOBa4gaamaaqaeabaGaemOzayMaemyAaKMaemyBa02aaSbaaSqaaiabg6da+aqabaaabeqab0GaeyyeIuoaaaa@3CC9@ is the fraction of targets whose models improve when choosing models using the FRST MQAP. We measure the average improvement in model quality as qim¯=1n∑qim
 MathType@MTEF@5@5@+=feaafiart1ev1aaatCvAUfKttLearuWrP9MDH5MBPbIqV92AaeXatLxBI9gBaebbnrfifHhDYfgasaacH8akY=wiFfYdH8Gipec8Eeeu0xXdbba9frFj0=OqFfea0dXdd9vqai=hGuQ8kuc9pgc9s8qqaq=dirpe0xb9q8qiLsFr0=vr0=vr0dc8meaabaqaciaacaGaaeqabaqabeGadaaakeaadaqdaaqaaiabdghaXjabdMgaPjabd2gaTbaacqGH9aqpdaWcaaqaaiabigdaXaqaaiabd6gaUbaadaaeabqaaiabdghaXjabdMgaPjabd2gaTbWcbeqab0GaeyyeIuoaaaa@3A8E@.

A summary of the results when selecting models according to the *frst *potential is given in Table 4.

#### 2.3.3 Spotting candidate targets with estimated improvement

Both model generation procedures *PVS *and *PVH *include the Arby default model in the ensemble of generated models. Therefore, for any target, model selection will only pick an alternative model, if a model with a score better than the Arby default exists. An indicator for this is

*ni*_*E,frst*_(*t*) = [*frst*(*s*_*E,fsrt*_(*t*)) <*frst*(*d*(*t*))].

The set of targets for which model selection proposes candidates with estimated improvement consists of *n*·ni¯
 MathType@MTEF@5@5@+=feaafiart1ev1aaatCvAUfKttLearuWrP9MDH5MBPbIqV92AaeXatLxBI9gBaebbnrfifHhDYfgasaacH8akY=wiFfYdH8Gipec8Eeeu0xXdbba9frFj0=OqFfea0dXdd9vqai=hGuQ8kuc9pgc9s8qqaq=dirpe0xb9q8qiLsFr0=vr0=vr0dc8meaabaqaciaacaGaaeqabaqabeGadaaakeaadaqdaaqaaiabd6gaUjabdMgaPbaaaaa@2F7D@ = ∑*ni *targets. On this candidate set, we denote the average improvement in model quality as qim¯=1n⋅ni¯∑tqim(t)
 MathType@MTEF@5@5@+=feaafiart1ev1aaatCvAUfKttLearuWrP9MDH5MBPbIqV92AaeXatLxBI9gBaebbnrfifHhDYfgasaacH8akY=wiFfYdH8Gipec8Eeeu0xXdbba9frFj0=OqFfea0dXdd9vqai=hGuQ8kuc9pgc9s8qqaq=dirpe0xb9q8qiLsFr0=vr0=vr0dc8meaabaqaciaacaGaaeqabaqabeGadaaakeaadaadaaqaaiabdghaXjabdMgaPjabd2gaTbaacqGH9aqpdaWcaaqaaiabigdaXaqaaiabd6gaUjabgwSixpaanaaabaGaemOBa4MaemyAaKgaaaaadaaeqaqaaiabdghaXjabdMgaPjabd2gaTjabcIcaOiabdsha0jabcMcaPaWcbaGaemiDaqhabeqdcqGHris5aaaa@440B@.

#### 2.3.4 Significance and coverage

The *fim*_> _and *qim *numbers exhibit a noticeable increase in model quality with respect to random selection of models (cf. fpt¯
 MathType@MTEF@5@5@+=feaafiart1ev1aaatCvAUfKttLearuWrP9MDH5MBPbIqV92AaeXatLxBI9gBaebbnrfifHhDYfgasaacH8akY=wiFfYdH8Gipec8Eeeu0xXdbba9frFj0=OqFfea0dXdd9vqai=hGuQ8kuc9pgc9s8qqaq=dirpe0xb9q8qiLsFr0=vr0=vr0dc8meaabaqaciaacaGaaeqabaqabeGadaaakeaadaqdaaqaaiabdAgaMjabdchaWjabdsha0baaaaa@30EC@_<_and qir¯
 MathType@MTEF@5@5@+=feaafiart1ev1aaatCvAUfKttLearuWrP9MDH5MBPbIqV92AaeXatLxBI9gBaebbnrfifHhDYfgasaacH8akY=wiFfYdH8Gipec8Eeeu0xXdbba9frFj0=OqFfea0dXdd9vqai=hGuQ8kuc9pgc9s8qqaq=dirpe0xb9q8qiLsFr0=vr0=vr0dc8meaabaqaciaacaGaaeqabaqabeGadaaakeaadaqdaaqaaiabdghaXjabdMgaPjabdkhaYbaaaaa@30F0@ values in Table 3).

More importantly, comparing models resulting from the selection process to the Arby default by applying a paired Wilcoxon signed rank test, we find for model generation procedure *PVS *that the models selected according to *frst *are significantly better than the Arby default (with a p-value of 0.002). For the model generation procedure *PVH*, the models selected with *frst *alone are neither significantly better nor worse than the default, demonstrating that it is hard to select better models when generating more low-quality models.

Selection of models constructed with model generation procedure *PVS *results in an average quality improvement of qim¯EPVS,frst
 MathType@MTEF@5@5@+=feaafiart1ev1aaatCvAUfKttLearuWrP9MDH5MBPbIqV92AaeXatLxBI9gBaebbnrfifHhDYfgasaacH8akY=wiFfYdH8Gipec8Eeeu0xXdbba9frFj0=OqFfea0dXdd9vqai=hGuQ8kuc9pgc9s8qqaq=dirpe0xb9q8qiLsFr0=vr0=vr0dc8meaabaqaciaacaGaaeqabaqabeGadaaakeaadaadaaqaaiabdghaXjabdMgaPjabd2gaTbaadaWgaaWcbaGaemyrau0aaSbaaWqaaiabdcfaqjabdAfawjabdofatjabcYcaSiabdAgaMjabdkhaYjabdohaZjabdsha0bqabaaaleqaaaaa@3C6B@ = 0.0031 and works better than selection of models constructed with model generation procedure *PVH *with an average quality improvement of qim¯EPVH,frst
 MathType@MTEF@5@5@+=feaafiart1ev1aaatCvAUfKttLearuWrP9MDH5MBPbIqV92AaeXatLxBI9gBaebbnrfifHhDYfgasaacH8akY=wiFfYdH8Gipec8Eeeu0xXdbba9frFj0=OqFfea0dXdd9vqai=hGuQ8kuc9pgc9s8qqaq=dirpe0xb9q8qiLsFr0=vr0=vr0dc8meaabaqaciaacaGaaeqabaqabeGadaaakeaadaadaaqaaiabdghaXjabdMgaPjabd2gaTbaadaWgaaWcbaGaemyrau0aaSbaaWqaaiabdcfaqjabdAfawjabdIeaijabcYcaSiabdAgaMjabdkhaYjabdohaZjabdsha0bqabaaaleqaaaaa@3C55@ = 0.00068.

For generating procedure *PVS*, the selection according to FRST suggests an alternative model for 51% of the targets; 53% of these suggested targets are improved according to *TM*-score. For generating procedure *PVH*, alternative models are suggested for 70% of the targets; 48% of these suggested targets are improved according to *TM*-score.

#### 2.3.5 Selection of high quality models using an SVM-based selection

Based on the FRST scores, an SVM was trained to choose high quality models as described in the Methods section. The values *fim*_*svm*_, *qim*_*svm*_, and *ni*_*svm *_are calculated analogously to the previously defined *fim*_*frst*_, *qim*_*frst*_, and *ni*_*frst*_, by replacing *frst *in these formulas with the negative SVM decision values.

#### 2.3.6 Significance and coverage of the SVM selection

**Figure 2:**
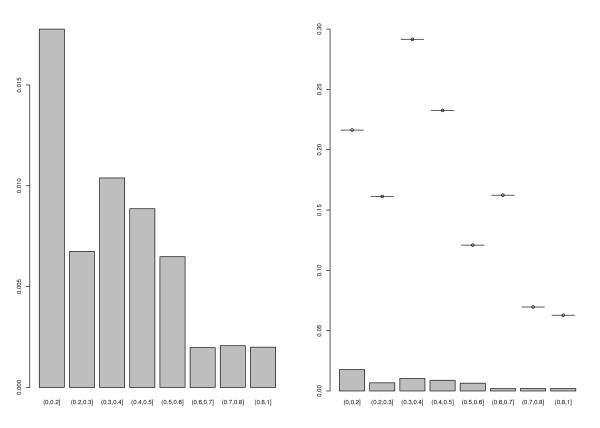
(Left) Average increase in *TM*-score, for ranges of difficulty. Targets are binned according to the *TM*-score of the default Arby model. Within each bin the average increase in quality *qim*is plotted. Bins are enumerated horizontally, the two outer bins were concatenated with their neighbors as each contained less than 100 target samples. Models are selected from *PVS *using the SVM. For comparison the average increase in quality obtained on this benchmark set by performing loop modeling is 0.003. (Right) Maximum increase in *TM*-score, for the same ranges of difficulty. The maximum increase in quality max *qim *within each bin is visualized as a line above the box representing the average increase (which is the same as on the left side, just the scale is different).

The results produced with selecting models according to the SVM decision values are summarized in Table 4. For *PVS*, an overview is given in Figure 2.

Compared to the FRST potentials, the ni¯
 MathType@MTEF@5@5@+=feaafiart1ev1aaatCvAUfKttLearuWrP9MDH5MBPbIqV92AaeXatLxBI9gBaebbnrfifHhDYfgasaacH8akY=wiFfYdH8Gipec8Eeeu0xXdbba9frFj0=OqFfea0dXdd9vqai=hGuQ8kuc9pgc9s8qqaq=dirpe0xb9q8qiLsFr0=vr0=vr0dc8meaabaqaciaacaGaaeqabaqabeGadaaakeaadaqdaaqaaiabd6gaUjabdMgaPbaaaaa@2F7D@_*sum *_are smaller (i.e. fewer targets were suggested for alteration, see Table 4). The SVM more effectively avoids changing models for the worse. This is visible in Figure 1 and also reflected by noticeably smaller average numbers of models with decreased quality (fim¯
 MathType@MTEF@5@5@+=feaafiart1ev1aaatCvAUfKttLearuWrP9MDH5MBPbIqV92AaeXatLxBI9gBaebbnrfifHhDYfgasaacH8akY=wiFfYdH8Gipec8Eeeu0xXdbba9frFj0=OqFfea0dXdd9vqai=hGuQ8kuc9pgc9s8qqaq=dirpe0xb9q8qiLsFr0=vr0=vr0dc8meaabaqaciaacaGaaeqabaqabeGadaaakeaadaqdaaqaaiabdAgaMjabdMgaPjabd2gaTbaaaaa@30D0@_<_values, see Table 4). The overall average improvement in *TM*-score model quality (qim¯
 MathType@MTEF@5@5@+=feaafiart1ev1aaatCvAUfKttLearuWrP9MDH5MBPbIqV92AaeXatLxBI9gBaebbnrfifHhDYfgasaacH8akY=wiFfYdH8Gipec8Eeeu0xXdbba9frFj0=OqFfea0dXdd9vqai=hGuQ8kuc9pgc9s8qqaq=dirpe0xb9q8qiLsFr0=vr0=vr0dc8meaabaqaciaacaGaaeqabaqabeGadaaakeaadaqdaaqaaiabdghaXjabdMgaPjabd2gaTbaaaaa@30E6@, *qim*) increases.

Applying a paired Wilcoxon signed rank test, we find for both generation procedures that the models selected by the SVM are significantly improved with respect to the Arby default (p-values below 10^-15^). The SVM selected models are also significantly improved with respect to the FRST selection process (with p-values below 10^-5^).

For generating procedure *PVS*, the SVM-based selection suggests an alternative model for 40% of the targets; 64% of these suggested targets are improved according to *TM*-score. For generating procedure *PVH*, alternative models are suggested for 58% of the targets; 61% of these suggested targets are improved according to *TM*-score.

## 3 Discussion

Jaroszewski et al. [9] show their method to produce significantly better alignments in about half of test cases, for a benchmark set of 742 protein pairs and make no statement regarding the likelihood of selecting such a solution from the ensemble of alternative alignments generated. To this end, they generate an average of 733 alignments per target-template pair with improved solutions in 34% of the test cases (average of 49 alignments). Our method is able to generate improved alignments for 59% of the test cases (*PVH*, Table 3) with only 13 alignments on average. The 55-fold decrease in the number of evaluated alignments compared to the method of Jaroszewski et al., while maintaining at least comparable increments in alignment quality, implies that we are exploring regions in the space of alignments that are densely populated with high-quality solutions, making the method practical for improving fully automated fold recognition servers such as Arby [23]. This is important when comparing our method to other approaches like ROBETTA [24], or the work of John and Sali [11] or Contreras-Moreira and coworkers [10], where improved model generation requires several orders of magnitude more alignments to be evaluated.

Improved alignments have to be selected from the ensemble of alternatives with an MQAP program in order to be useful. Neither Jaroszewski [9] nor Chivian [24] make quantitative statements about the selection of improved solutions. The results of John and Sali or *in silico *recombination are not directly comparable, as they generate and select the solutions iteratively. Our data show that the selection of improved alignments is a difficult task. A random selector would actually deteriorate the overall performance of the method. Generating more models does not necessarily help the selection process. Especially if more models below default quality are generated (as with generating procedure *PVH*), avoiding to select worse models is more difficult. Thus the error rate can increase and the overall performance can decrease if more low-quality models are generated.

Here we show that the proposed protocol, including model generation and SVM-based selection, significantly improves model quality (p-values below 10^-15 ^using a Wilcoxon signed rank test). With the model generation procedure *PVS*, using the SVM-based selection the proposed method achieves a close to optimal average *TM*-score improvement of 0.016 and a maximal observed increase in *TM*-quality of max *qim *= 0.29 This has to be related to typical fold recognition targets, where the *TM*-scores for large portions of the predictions lie in the range of 0.1 to 0.4 for hard targets and from 0.4 to 0.8 for easier targets.

To emphasize the relevance of the proposed method in practical use, we compare the quality improvement of the proposed protocol with the quality gain obtained by loop modeling alone. The average quality increase in *TM*-score incurred by using our protocol amounts to 0.016, which is a factor of five above the quality gain obtained by loop modeling alone 0.003. The quality increase for our protocol is computed as the difference between the *TM*-scores of the MQAP selection from varied models with modeled loops minus the Arby default with loops modeled. The model quality obtained by loop modeling alone is computed as the difference of the *TM*-score of the Arby default with loops modeled minus that of the Arby default without loops modeled (see additional file supplement).

## 4 Conclusion

We have presented an approach for improving structure prediction models that goes in a different direction from the one recently proposed by Pettitt et al. [12]. Whereas they have evaluated the possibility to choose better templates with an MQAP program, we show that it is possible to generate and select better alignments for a fixed template with an MQAP program. The two approaches can be combined and will improve automated servers such as Arby.

As this seems a promising approach in a competitive field, we will continue to work on the topic in two directions: First, generation of models with a high likelihood of improving the quality and second improving the selection process. For the latter, the numbers on the SVM performance clearly indicate that the current linear combination of the partial potentials in FRST can be improved.

## 5 Methods

### 5.1 Protocol

#### 5.1.1 Alternative alignments and models

The 3D protein structure model that we construct for a target protein is based on an alignment with a template structure. The method described here is independent of the strategy for template identification. With a given target and template as input, we compute a *default *alignment using profile-profile alignment with log-average scoring and parameters as tuned for the Arby server [22,23]. Namely these parameters are: substitution matrix Blosum62, gap insertion 14.7, gap extension 0.37, and a relative weight of secondary structure to sequence information of 0.24.

In addition to the Arby default alignment, we propose two procedures (*PVS, PVH *)for generating alternative alignments for a target in analogy to the parametric approach of Jaroszewski et al. [9]. The alternatives are computed by a global profile-profile alignment method, using parameters multiplied with a factor varied inside the range from a lower to an upper bound. The parameters varied are gap-insertion, gap-extension, and the relative weights of amino-acid and secondary structure profiles. The two procedures differ with respect to the ranges of the factors. Each procedure reports alignments that occur multiply for different parameter settings only one time, resulting in an ensemble of distinct alignments.

For each alignment a model is built as follows. Loop modeling of insertions and deletions is performed, using the LOBO program [25]. Conserved (i.e. identical) residues and their side chains are copied from the template structure. The non-conserved residues and their side chains are positioned and optimized by SCWRL3.0 [26].

#### 5.1.2 Model scores

The quality of the model is then estimated using the FRST MQAP program [13], which computes four potentials, namely a residue-specific all-atom distance potential [27] (*rapdf*), a solvation potential (*solv*), a hydrogen bonding potential (*hydb*), and a torsion angle potential (*tors*). These four potentials are linearly combined into the *frst *energy score (with factors 2.5, 500.0, -50.0, and 350.0, respectively [13]). This leaves us with the *frst *score as an estimate of the quality of each constructed model.

We can select the best alignment-based model for each target, by choosing the model with the lowest energy score according to the *frst *potential. These selected models are potentially improvements over the default model (constructed according to the default alignment). In the supplement we additionally analyze selection according to the partial contributions *rapdf, solv, hydb*, and *tors *of the *frst *potential (see additional file supplement). The FRST MQAP program places a strong emphasis on the torsion angle component [13]. Since each residue can either increase or decrease the overall score, there is no correlation between the number of gaps in a model and the overall score.

For 95% of the targets in the benchmark set of this paper, the FRST MQAP can distinguish the native structures from the Arby default models. Similarly, the performance of FRST on selecting the native structure from the models generated with procedures *PVS *and *PVH is *95% and 94%, respectively.

#### 5.1.3 Model quality evaluation

If, additionally, the native structure of the target is known, using the model evaluation programs GDT [28], MaxSub [17], and TM-score [18], we can compute scores (*GDT, MS, TM*), reflecting the "real" quality of the model in terms of structural similarity between model structure and target structure.

In general the quality measures *GDT*, *MS*, and *TM *correlate well: The correlation coefficients between quality measures for all models produced are cor_*GDT,MS *_= 0.99, cor_*GDT,TM *_= 0.93, and cor_*MS,TM *_= 0.93 (see supplement, Table 1). Overall the analysis yields similar results for all three quality measures. As the *TM*-score has the advantage of being independent of the size of the protein, we restrict our presentation to the analysis of the *TM*-score.

Overall, a moderate negative correlation cor_*TM,frst *_= -0.43 of the quality measure *TM*-score with the *frst *score can be observed. It has to be pointed out that the correlation of the *frst *score across all targets is not as relevant as its selection capabilities per target.

#### 5.1.4 Combining MQAP partial potentials using a support vector machine

We train a Support Vector Machine (SVM) for selecting models with higher *TM*-score than the *TM*-score of the default model. The binary labels used for each model are *TM*-score-increase and *TM*-score-decrease with respect to the default Arby model. As features we use the *frst, rapdf, solv, hydb, tors *values of each model and the corresponding default model as well as the differences of these scores between model and default. For each target, the best model is selected based on the SVM decision value [29]. Models with a negative SVM decision value remain unchanged with respect to the Arby default. As SVM implementation, the R package e1071 [30] based on libsvm [31] is employed. As parameter tuning showed only negligible changes in classification accuracy, standard parameters and a radial basis function kernel are used.

### 5.2 Benchmarking

#### 5.2.1 Dataset of targets and templates

For the validation of our approach, the improvement of the proposed models over the default Arby models was evaluated. Target sequences were taken from a representative set of SCOP 1.65 domains [32] with at most 40% sequence identity as provided by the Astral compendium [33,34]. As a basis for the alternative models, in this study, one template was chosen for each target: With log-average scoring and default parameters as listed in Table 1[22,23], the target was compared against the rest of the domains in the Astral 40% set and the top ranking hit was chosen as template. Our analysis was restricted to targets which have a template with at least 25% sequence identity, evaluating the proposed method for targets from the homologous fold recognition category. These criteria specify 1765 targets, each with one template. For 153 (8.7%) of these 1765 targets, some of the necessary computations failed. We excluded those targets, which leaves us with *n *= 1612 targets, for which we have all relevant scores available.

#### 5.2.2 Cross- validation of SVM-based selection

The training and validation of the support vector machine is performed using five-fold cross-validation. In order to ensure that there are no models for the same target in the training as well as in the testing set, during the cross-validation successively models for one fifth of the targets (not: one fifth of the models) are removed from the training set and used for testing.

As the pairwise sequence identity between targets is below 40% according to selection criteria it is guaranteed that models in the test and training sets are sufficiently distinct.

In order to assess the effect of the choice of *k *in *k*-fold cross-validation, a ten-fold cross-validation was also performed, yielding results identical to one digit precision in Table 4 (data not shown, the figures in the article refer to *k *= 5).

## Abbreviations

*a*_0_(*t*) Arby default model for targets *t*

*E*(*t*) Set of models constructed for targets *t *according to model generation procedure *i *∈ {0,1,2}

*TM *Model quality evaluation measure as computed with the TM-score program

*GDT *Model quality evaluation measure as computed with the LGA program

*MS *Model quality evaluation measure as computed with the MaxSub program

[*x*] Iverson bracket

x¯
 MathType@MTEF@5@5@+=feaafiart1ev1aaatCvAUfKttLearuWrP9MDH5MBPbIqV92AaeXatLxBI9gBaebbnrfifHhDYfgasaacH8akY=wiFfYdH8Gipec8Eeeu0xXdbba9frFj0=OqFfea0dXdd9vqai=hGuQ8kuc9pgc9s8qqaq=dirpe0xb9q8qiLsFr0=vr0=vr0dc8meaabaqaciaacaGaaeqabaqabeGadaaakeaadaqdaaqaaiabdIha4baaaaa@2E36@ Average of *x *over all targets

*x*Average of *x *over targets suggested by the selection procedure

*fpt*_*E*,>_(*t*) Relative **f**equency of models **p**er **t**arget with *TM*-score above Arby default

*qir*_*E*_(*t*) **Q**uality **i**mprovement when choosing **r**andomly

*fb*_*E*_(*t*) Indicator whether better model exists per target

fb¯
 MathType@MTEF@5@5@+=feaafiart1ev1aaatCvAUfKttLearuWrP9MDH5MBPbIqV92AaeXatLxBI9gBaebbnrfifHhDYfgasaacH8akY=wiFfYdH8Gipec8Eeeu0xXdbba9frFj0=OqFfea0dXdd9vqai=hGuQ8kuc9pgc9s8qqaq=dirpe0xb9q8qiLsFr0=vr0=vr0dc8meaabaqaciaacaGaaeqabaqabeGadaaakeaadaqdaaqaaiabdAgaMjabdkgaIbaaaaa@2F5F@_*E*_(*t*) **R**elative **f**requency of targets for which a **b**etter model exists

*qib*_*E*_(*t*) **Q**uality **i**mprovement which is theoretically the **b**est possible

*s*_*E*,*frst*_(*t*) **S**elected model per target

*fim*_*E*,*frst*,>_(*t*) Indicator whether selected model is improved in real quality

fim¯
 MathType@MTEF@5@5@+=feaafiart1ev1aaatCvAUfKttLearuWrP9MDH5MBPbIqV92AaeXatLxBI9gBaebbnrfifHhDYfgasaacH8akY=wiFfYdH8Gipec8Eeeu0xXdbba9frFj0=OqFfea0dXdd9vqai=hGuQ8kuc9pgc9s8qqaq=dirpe0xb9q8qiLsFr0=vr0=vr0dc8meaabaqaciaacaGaaeqabaqabeGadaaakeaadaqdaaqaaiabdAgaMjabdMgaPjabd2gaTbaaaaa@30D0@_*E*,*frst*,>_(*t*) Relative **f**requency of selected models with **i**ncreased *TM*-score **m**odel quality

*qim*_*E,frst*_(*t*) Measure of **q**uality **i**mprovement with respect to default Arby **m**odel

ni¯
 MathType@MTEF@5@5@+=feaafiart1ev1aaatCvAUfKttLearuWrP9MDH5MBPbIqV92AaeXatLxBI9gBaebbnrfifHhDYfgasaacH8akY=wiFfYdH8Gipec8Eeeu0xXdbba9frFj0=OqFfea0dXdd9vqai=hGuQ8kuc9pgc9s8qqaq=dirpe0xb9q8qiLsFr0=vr0=vr0dc8meaabaqaciaacaGaaeqabaqabeGadaaakeaadaqdaaqaaiabd6gaUjabdMgaPbaaaaa@2F7D@ Relative frequency of targets for which a selection procedure suggests **i**mproved models

The indicator functions are constructed to count the relative frequencies. They draw their names from the respective relative frequencies.

## 7 Authors' contributions

S.C.E.T, S.T., and I.S. conceived the experiment, I.S. and S.T. performed the experiment, I.S., O.S., and T.L. analyzed the results, and I.S., T.L., and S.C.E.T. wrote the final manuscript, which all authors have approved.

## Supplementary Material

Additional File 1 Additional statistical analysis of partial potentials and of models with and without loop modeling. Some additional material, analysising partial potentials and analyzing the behaviour of the protocol when using models with our without loop modeling performed. Click here for file

## Figures and Tables

**Table 1:** The parameters used in the different model generating procedures.

Model Generating Procedure	Description
Arby default	Generates the default Arby models [23], exactly one for each target. Default parameters are: gap insertion 14.7, gap extension 0.37, substitution matrix Blosum62, Henikoff position-specific sequence weighting, relative weight of secondary structure is 0.24.
*PVS*	Generates models resulting from alignments with parameters multiplied by a factor varying from 0.5 to 1.5 with a step width of 0.25. This results in an ensemble of alternative models for each target.
*PVH*	Generates models resulting from alignments with parameters multiplied by a factor from 0.2 to 2.4 with a step width of 0.2. This results in an ensemble of alternative models for each target.

**Table 2:** Summary statistics for the model generation procedures.

Model Generation Procedure	Number of Models per Target *t*				Overall Number of Models (∑_*t*_|*E*(*t*)|)
	min	median	mean	max	
Arby default	1	1.0	1.0	1	1612
*PVS*	1	3.0	4.3	28	6980
*PVH*	1	7.0	12.6	136	20234

**Table 3:** How good are the generated models? Description of the distributions of the *TM*-score quality. fpt¯
 MathType@MTEF@5@5@+=feaafiart1ev1aaatCvAUfKttLearuWrP9MDH5MBPbIqV92AaeXatLxBI9gBaebbnrfifHhDYfgasaacH8akY=wiFfYdH8Gipec8Eeeu0xXdbba9frFj0=OqFfea0dXdd9vqai=hGuQ8kuc9pgc9s8qqaq=dirpe0xb9q8qiLsFr0=vr0=vr0dc8meaabaqaciaacaGaaeqabaqabeGadaaakeaadaqdaaqaaiabdAgaMjabdchaWjabdsha0baaaaa@30EC@_<_and fpt¯
 MathType@MTEF@5@5@+=feaafiart1ev1aaatCvAUfKttLearuWrP9MDH5MBPbIqV92AaeXatLxBI9gBaebbnrfifHhDYfgasaacH8akY=wiFfYdH8Gipec8Eeeu0xXdbba9frFj0=OqFfea0dXdd9vqai=hGuQ8kuc9pgc9s8qqaq=dirpe0xb9q8qiLsFr0=vr0=vr0dc8meaabaqaciaacaGaaeqabaqabeGadaaakeaadaqdaaqaaiabdAgaMjabdchaWjabdsha0baaaaa@30EC@_> _are the relative frequencies of models per target with a *TM*-score below and above Arby default, respectively, qir¯
 MathType@MTEF@5@5@+=feaafiart1ev1aaatCvAUfKttLearuWrP9MDH5MBPbIqV92AaeXatLxBI9gBaebbnrfifHhDYfgasaacH8akY=wiFfYdH8Gipec8Eeeu0xXdbba9frFj0=OqFfea0dXdd9vqai=hGuQ8kuc9pgc9s8qqaq=dirpe0xb9q8qiLsFr0=vr0=vr0dc8meaabaqaciaacaGaaeqabaqabeGadaaakeaadaqdaaqaaiabdghaXjabdMgaPjabdkhaYbaaaaa@30F0@ is the improvement in *TM*-score when choosing models randomly. fb¯
 MathType@MTEF@5@5@+=feaafiart1ev1aaatCvAUfKttLearuWrP9MDH5MBPbIqV92AaeXatLxBI9gBaebbnrfifHhDYfgasaacH8akY=wiFfYdH8Gipec8Eeeu0xXdbba9frFj0=OqFfea0dXdd9vqai=hGuQ8kuc9pgc9s8qqaq=dirpe0xb9q8qiLsFr0=vr0=vr0dc8meaabaqaciaacaGaaeqabaqabeGadaaakeaadaqdaaqaaiabdAgaMjabdkgaIbaaaaa@2F5F@ is the relative frequency of targets for which a better model exists, qib¯
 MathType@MTEF@5@5@+=feaafiart1ev1aaatCvAUfKttLearuWrP9MDH5MBPbIqV92AaeXatLxBI9gBaebbnrfifHhDYfgasaacH8akY=wiFfYdH8Gipec8Eeeu0xXdbba9frFj0=OqFfea0dXdd9vqai=hGuQ8kuc9pgc9s8qqaq=dirpe0xb9q8qiLsFr0=vr0=vr0dc8meaabaqaciaacaGaaeqabaqabeGadaaakeaadaqdaaqaaiabdghaXjabdMgaPjabdkgaIbaaaaa@30D0@ is the best theoretically possible improvement for the given ensemble of models.

Model Generation Procedure	fpt¯ MathType@MTEF@5@5@+=feaafiart1ev1aaatCvAUfKttLearuWrP9MDH5MBPbIqV92AaeXatLxBI9gBaebbnrfifHhDYfgasaacH8akY=wiFfYdH8Gipec8Eeeu0xXdbba9frFj0=OqFfea0dXdd9vqai=hGuQ8kuc9pgc9s8qqaq=dirpe0xb9q8qiLsFr0=vr0=vr0dc8meaabaqaciaacaGaaeqabaqabeGadaaakeaadaqdaaqaaiabdAgaMjabdchaWjabdsha0baaaaa@30EC@_<_	fpt¯ MathType@MTEF@5@5@+=feaafiart1ev1aaatCvAUfKttLearuWrP9MDH5MBPbIqV92AaeXatLxBI9gBaebbnrfifHhDYfgasaacH8akY=wiFfYdH8Gipec8Eeeu0xXdbba9frFj0=OqFfea0dXdd9vqai=hGuQ8kuc9pgc9s8qqaq=dirpe0xb9q8qiLsFr0=vr0=vr0dc8meaabaqaciaacaGaaeqabaqabeGadaaakeaadaqdaaqaaiabdAgaMjabdchaWjabdsha0baaaaa@30EC@_>_	qir¯ MathType@MTEF@5@5@+=feaafiart1ev1aaatCvAUfKttLearuWrP9MDH5MBPbIqV92AaeXatLxBI9gBaebbnrfifHhDYfgasaacH8akY=wiFfYdH8Gipec8Eeeu0xXdbba9frFj0=OqFfea0dXdd9vqai=hGuQ8kuc9pgc9s8qqaq=dirpe0xb9q8qiLsFr0=vr0=vr0dc8meaabaqaciaacaGaaeqabaqabeGadaaakeaadaqdaaqaaiabdghaXjabdMgaPjabdkhaYbaaaaa@30F0@	fb¯ MathType@MTEF@5@5@+=feaafiart1ev1aaatCvAUfKttLearuWrP9MDH5MBPbIqV92AaeXatLxBI9gBaebbnrfifHhDYfgasaacH8akY=wiFfYdH8Gipec8Eeeu0xXdbba9frFj0=OqFfea0dXdd9vqai=hGuQ8kuc9pgc9s8qqaq=dirpe0xb9q8qiLsFr0=vr0=vr0dc8meaabaqaciaacaGaaeqabaqabeGadaaakeaadaqdaaqaaiabdAgaMjabdkgaIbaaaaa@2F5F@	qib¯ MathType@MTEF@5@5@+=feaafiart1ev1aaatCvAUfKttLearuWrP9MDH5MBPbIqV92AaeXatLxBI9gBaebbnrfifHhDYfgasaacH8akY=wiFfYdH8Gipec8Eeeu0xXdbba9frFj0=OqFfea0dXdd9vqai=hGuQ8kuc9pgc9s8qqaq=dirpe0xb9q8qiLsFr0=vr0=vr0dc8meaabaqaciaacaGaaeqabaqabeGadaaakeaadaqdaaqaaiabdghaXjabdMgaPjabdkgaIbaaaaa@30D0@
*PVS*	0.36	0.22	-0.013	0.47	0.019
*PVH*	0.51	0.26	-0.031	0.59	0.026

**Table 4:** How well does model selection work? Description of distributions when selecting models according to the FRST potentials and the SVM. ni¯
 MathType@MTEF@5@5@+=feaafiart1ev1aaatCvAUfKttLearuWrP9MDH5MBPbIqV92AaeXatLxBI9gBaebbnrfifHhDYfgasaacH8akY=wiFfYdH8Gipec8Eeeu0xXdbba9frFj0=OqFfea0dXdd9vqai=hGuQ8kuc9pgc9s8qqaq=dirpe0xb9q8qiLsFr0=vr0=vr0dc8meaabaqaciaacaGaaeqabaqabeGadaaakeaadaqdaaqaaiabd6gaUjabdMgaPbaaaaa@2F7D@ is the relative frequency of targets for which a selection procedure suggests improved models. fim¯
 MathType@MTEF@5@5@+=feaafiart1ev1aaatCvAUfKttLearuWrP9MDH5MBPbIqV92AaeXatLxBI9gBaebbnrfifHhDYfgasaacH8akY=wiFfYdH8Gipec8Eeeu0xXdbba9frFj0=OqFfea0dXdd9vqai=hGuQ8kuc9pgc9s8qqaq=dirpe0xb9q8qiLsFr0=vr0=vr0dc8meaabaqaciaacaGaaeqabaqabeGadaaakeaadaqdaaqaaiabdAgaMjabdMgaPjabd2gaTbaaaaa@30D0@_<_, fim¯
 MathType@MTEF@5@5@+=feaafiart1ev1aaatCvAUfKttLearuWrP9MDH5MBPbIqV92AaeXatLxBI9gBaebbnrfifHhDYfgasaacH8akY=wiFfYdH8Gipec8Eeeu0xXdbba9frFj0=OqFfea0dXdd9vqai=hGuQ8kuc9pgc9s8qqaq=dirpe0xb9q8qiLsFr0=vr0=vr0dc8meaabaqaciaacaGaaeqabaqabeGadaaakeaadaqdaaqaaiabdAgaMjabdMgaPjabd2gaTbaaaaa@30D0@_=_, and fim¯
 MathType@MTEF@5@5@+=feaafiart1ev1aaatCvAUfKttLearuWrP9MDH5MBPbIqV92AaeXatLxBI9gBaebbnrfifHhDYfgasaacH8akY=wiFfYdH8Gipec8Eeeu0xXdbba9frFj0=OqFfea0dXdd9vqai=hGuQ8kuc9pgc9s8qqaq=dirpe0xb9q8qiLsFr0=vr0=vr0dc8meaabaqaciaacaGaaeqabaqabeGadaaakeaadaqdaaqaaiabdAgaMjabdMgaPjabd2gaTbaaaaa@30D0@_>_, are the relative frequencies of selected models with decreased, equal, or increased *TM*-score quality, respectively. min *qim *and max *qim *are the minimal and maximal quality improvements achieved per target. qim¯
 MathType@MTEF@5@5@+=feaafiart1ev1aaatCvAUfKttLearuWrP9MDH5MBPbIqV92AaeXatLxBI9gBaebbnrfifHhDYfgasaacH8akY=wiFfYdH8Gipec8Eeeu0xXdbba9frFj0=OqFfea0dXdd9vqai=hGuQ8kuc9pgc9s8qqaq=dirpe0xb9q8qiLsFr0=vr0=vr0dc8meaabaqaciaacaGaaeqabaqabeGadaaakeaadaqdaaqaaiabdghaXjabdMgaPjabd2gaTbaaaaa@30E6@ is the average quality improvement over all targets. *qim*is the average quality improvement for the targets that the selection procedure suggests improved models for.

	*Model Generation Procedure PVS*, all measures for *TM*							
e	ni¯ MathType@MTEF@5@5@+=feaafiart1ev1aaatCvAUfKttLearuWrP9MDH5MBPbIqV92AaeXatLxBI9gBaebbnrfifHhDYfgasaacH8akY=wiFfYdH8Gipec8Eeeu0xXdbba9frFj0=OqFfea0dXdd9vqai=hGuQ8kuc9pgc9s8qqaq=dirpe0xb9q8qiLsFr0=vr0=vr0dc8meaabaqaciaacaGaaeqabaqabeGadaaakeaadaqdaaqaaiabd6gaUjabdMgaPbaaaaa@2F7D@	_<_	fim¯ MathType@MTEF@5@5@+=feaafiart1ev1aaatCvAUfKttLearuWrP9MDH5MBPbIqV92AaeXatLxBI9gBaebbnrfifHhDYfgasaacH8akY=wiFfYdH8Gipec8Eeeu0xXdbba9frFj0=OqFfea0dXdd9vqai=hGuQ8kuc9pgc9s8qqaq=dirpe0xb9q8qiLsFr0=vr0=vr0dc8meaabaqaciaacaGaaeqabaqabeGadaaakeaadaqdaaqaaiabdAgaMjabdMgaPjabd2gaTbaaaaa@30D0@_=_	fim¯ MathType@MTEF@5@5@+=feaafiart1ev1aaatCvAUfKttLearuWrP9MDH5MBPbIqV92AaeXatLxBI9gBaebbnrfifHhDYfgasaacH8akY=wiFfYdH8Gipec8Eeeu0xXdbba9frFj0=OqFfea0dXdd9vqai=hGuQ8kuc9pgc9s8qqaq=dirpe0xb9q8qiLsFr0=vr0=vr0dc8meaabaqaciaacaGaaeqabaqabeGadaaakeaadaqdaaqaaiabdAgaMjabdMgaPjabd2gaTbaaaaa@30D0@_>_	min *qim*	max *qim*	qim¯ MathType@MTEF@5@5@+=feaafiart1ev1aaatCvAUfKttLearuWrP9MDH5MBPbIqV92AaeXatLxBI9gBaebbnrfifHhDYfgasaacH8akY=wiFfYdH8Gipec8Eeeu0xXdbba9frFj0=OqFfea0dXdd9vqai=hGuQ8kuc9pgc9s8qqaq=dirpe0xb9q8qiLsFr0=vr0=vr0dc8meaabaqaciaacaGaaeqabaqabeGadaaakeaadaqdaaqaaiabdghaXjabdMgaPjabd2gaTbaaaaa@30E6@	*qim*
*frst*	0.51	0.23	0.50	0.27	-0.51	0.35	0.0016	0.0031
SVM	0.40	0.14	0.61	0.25	-0.21	0.29	0.0064	0.0160
	*Model Generation Procedure PVH*, all measures for *TM*							
e	ni¯ MathType@MTEF@5@5@+=feaafiart1ev1aaatCvAUfKttLearuWrP9MDH5MBPbIqV92AaeXatLxBI9gBaebbnrfifHhDYfgasaacH8akY=wiFfYdH8Gipec8Eeeu0xXdbba9frFj0=OqFfea0dXdd9vqai=hGuQ8kuc9pgc9s8qqaq=dirpe0xb9q8qiLsFr0=vr0=vr0dc8meaabaqaciaacaGaaeqabaqabeGadaaakeaadaqdaaqaaiabd6gaUjabdMgaPbaaaaa@2F7D@	fim¯ MathType@MTEF@5@5@+=feaafiart1ev1aaatCvAUfKttLearuWrP9MDH5MBPbIqV92AaeXatLxBI9gBaebbnrfifHhDYfgasaacH8akY=wiFfYdH8Gipec8Eeeu0xXdbba9frFj0=OqFfea0dXdd9vqai=hGuQ8kuc9pgc9s8qqaq=dirpe0xb9q8qiLsFr0=vr0=vr0dc8meaabaqaciaacaGaaeqabaqabeGadaaakeaadaqdaaqaaiabdAgaMjabdMgaPjabd2gaTbaaaaa@30D0@_<_	fim¯ MathType@MTEF@5@5@+=feaafiart1ev1aaatCvAUfKttLearuWrP9MDH5MBPbIqV92AaeXatLxBI9gBaebbnrfifHhDYfgasaacH8akY=wiFfYdH8Gipec8Eeeu0xXdbba9frFj0=OqFfea0dXdd9vqai=hGuQ8kuc9pgc9s8qqaq=dirpe0xb9q8qiLsFr0=vr0=vr0dc8meaabaqaciaacaGaaeqabaqabeGadaaakeaadaqdaaqaaiabdAgaMjabdMgaPjabd2gaTbaaaaa@30D0@_=_	fim¯ MathType@MTEF@5@5@+=feaafiart1ev1aaatCvAUfKttLearuWrP9MDH5MBPbIqV92AaeXatLxBI9gBaebbnrfifHhDYfgasaacH8akY=wiFfYdH8Gipec8Eeeu0xXdbba9frFj0=OqFfea0dXdd9vqai=hGuQ8kuc9pgc9s8qqaq=dirpe0xb9q8qiLsFr0=vr0=vr0dc8meaabaqaciaacaGaaeqabaqabeGadaaakeaadaqdaaqaaiabdAgaMjabdMgaPjabd2gaTbaaaaa@30D0@_>_	min *qim*	max *qim*	qim¯ MathType@MTEF@5@5@+=feaafiart1ev1aaatCvAUfKttLearuWrP9MDH5MBPbIqV92AaeXatLxBI9gBaebbnrfifHhDYfgasaacH8akY=wiFfYdH8Gipec8Eeeu0xXdbba9frFj0=OqFfea0dXdd9vqai=hGuQ8kuc9pgc9s8qqaq=dirpe0xb9q8qiLsFr0=vr0=vr0dc8meaabaqaciaacaGaaeqabaqabeGadaaakeaadaqdaaqaaiabdghaXjabdMgaPjabd2gaTbaaaaa@30E6@	*qim*

*frst*	0.70	0.35	0.31	0.34	-0.43	0.29	0.00047	0.00068
SVM	0.58	0.22	0.43	0.35	-0.27	0.35	0.00774	0.01339
