# Supplementary material for: Improving the quality of protein structure models by selecting from alignment alternatives
Source: BMC Bioinformatics. 2006 Jul 27;7:364. doi: 10.1186/1471-2105-7-364 (PMC1579234; doi:10.1186/1471-2105-7-364)
Supplement: Additional File 1 — Additional statistical analysis of partial potentials and of models with and without loop modeling. Some additional material, analysising partial potentials and analyzing the behaviour of the protocol when using models with our without loop modeling performed. [file 1471-2105-7-364-S1.pdf]

Supplementary Material
Improving the Quality of Protein Structure Models by Selecting from Alignment Alternatives

I. Sommer*, S. Toppo, O. Sander, T. Lengauer, S.C.E. Tosatto

Email: sommer@mpi-sb.mpg.de;

*Corresponding author

All-Against-All Correlation

Table 1 lists the correlation coefficients of the FRST potentials *frst*, *rapdf*, *solv*, *hydb*, *tors* with the model quality scores *GDT*, *MS*, *TM*.

As a general trend, a moderate negative correlation can be observed between the FRST partial potentials and the quality measures. As was pointed out for the *frst* potential in the main manuscript, the correlation of the FRST partial potentials across all targets are not as relevant as their selection capabilities per target.

Models with and without Loop Modeling

This study was performed for models with and models without loop modeling in parallel. The numbers in the main manuscript always refer to the models for which loop modeling was performed. Here, in the supplement we provide both.

In order to provide a fair comparison of the method proposed, although the Arby webserver does not provide loop modeling at the moment, we use default models with loop modeling performed for the analysis of the loop modeling case.

The overall tendency of the results remains the same, whether or not loop modeling is performed. The average difference in *TM*-score between a default model with loop modeling and a default model without loop modeling is 0.003.

Table 2 summarizes the results of model generation with and without loop modeling.

Analysis per Target

In section 2.3.1 of the main manuscript, the selection of models according to the *frst* potential is evaluated. Analogously, the partial potentials *rapdf*, *solv*, *hydb*, and *tors* of the FRST method can be employed for model selection, by using these values instead of *frst* in the respective formulas. The results of this process are listed in Table 3.

Percentage of Noticeably Improved Cases

We compute the average number of cases where the TM is improved more than 0.05 as

$$\underline{ci}_{E,q,e,>0.05} = \frac{1}{n \cdot \overline{ni}} \sum_{t \in T} \left[qim_{E,q,e}(t) > 0.05 \right]$$

or worsened more than -0.05 as

$$\underline{ci}_{E,q,e,<-0.05} = \frac{1}{n \cdot \overline{ni}} \sum_{t \in T} \left[qim_{E,q,e}(t) < -0.05 \right]$$

These numbers are also summarized in Table 3.

	frst	*rapdf*	*solv*	*hydb*	*tors*	*TM*	*MS*	*GDT*
frst	1.00	0.02	0.73	-0.34	0.95	-0.43	-0.50	-0.52
rapdf	0.02	1.00	0.30	0.86	-0.14	-0.31	-0.11	-0.08
solv	0.73	0.30	1.00	0.06	0.53	-0.51	-0.46	-0.47
hydb	-0.34	0.86	0.06	1.00	-0.40	-0.15	0.10	0.13
tors	0.95	-0.14	0.53	-0.40	1.00	-0.33	-0.43	-0.44
TM	-0.43	-0.31	-0.51	-0.15	-0.33	1.00	0.93	0.93
MS	-0.50	-0.11	-0.46	0.10	-0.43	0.93	1.00	0.99
GDT	-0.52	-0.08	-0.47	0.13	-0.44	0.93	0.99	1.00

Table 1: Correlation coefficients between FRST potentials and quality scores GDT, *MS*, and *TM* across all models $E(t)$, for all targets t and models created with PVS and PVH joined.

Model Generation Procedure	$\overline{fr}_<$	$\overline{fr}_>$	$\overline{qir}$	$\overline{fb}$	$\overline{qib}$
PVS, w/o loop modeling	0.37	0.21	-0.013	0.45	0.018
PVH, w/o loop modeling	0.54	0.23	-0.032	0.56	0.023
PVS, with loop modeling	0.36	0.22	-0.013	0.47	0.019
PVH, with loop modeling	0.51	0.26	-0.031	0.59	0.026

Table 2: Description of the distributions of the *TM*-score quality behavior. $\overline{fr}_<$ and $\overline{fr}_>$ are the relative frequencies of models per target with a *TM*-score below and above Arby default, respectively. $\overline{qir}$ is the improvement in *TM*-score when choosing models randomly. $\overline{fb}$ is the relative frequency of targets for which a better model exists. $\overline{qib}$ is the best theoretically possible improvement for the given ensemble of models.

PVS, all Measures for TM-score, no loop modeling performed

e	$\overline{ni}$	$\overline{fm}_{<}$	$\overline{fm}_{=}$	$\overline{fm}_{>}$	min qim	max qim	$\overline{qim}$	$\underline{qim}$	$\underline{ci}_{< -0.05}$	$\underline{ci}_{> 0.05}$
frst	0.52	0.25	0.49	0.26	-0.54	0.24	0.0011	0.0021	0.101	0.125
rapdf	0.48	0.19	0.53	0.28	-0.25	0.31	0.0068	0.0142	0.063	0.174
solv	0.53	0.25	0.48	0.27	-0.44	0.27	0.0025	0.0047	0.096	0.151
hydb	0.45	0.31	0.55	0.14	-0.46	0.25	-0.0182	-0.0404	0.356	0.091
tors	0.53	0.29	0.48	0.23	-0.54	0.24	-0.0053	-0.0098	0.147	0.094
SVM	0.40	0.14	0.60	0.25	-0.19	0.25	0.0069	0.0170	0.035	0.164

PVH, all Measures for TM-score, no loop modeling performed

e	$\overline{ni}$	$\overline{fm}_{<}$	$\overline{fm}_{=}$	$\overline{fm}_{>}$	min qim	max qim	$\overline{qim}$	$\underline{qim}$	$\underline{ci}_{< -0.05}$	$\underline{ci}_{> 0.05}$
frst	0.70	0.38	0.31	0.31	-0.63	0.24	-0.0032	-0.0047	0.115	0.096
rapdf	0.62	0.27	0.39	0.34	-0.30	0.32	0.0062	0.0100	0.068	0.146
solv	0.70	0.38	0.31	0.31	-0.44	0.32	-0.0019	-0.0027	0.119	0.103
hydb	0.62	0.48	0.38	0.14	-0.52	0.32	-0.0603	-0.0974	0.558	0.073
tors	0.70	0.42	0.30	0.27	-0.64	0.24	-0.0110	-0.0157	0.165	0.075
SVM	0.55	0.22	0.45	0.32	-0.23	0.32	0.0072	0.0130	0.045	0.139

PVS, all Measures for TM-score, loop modeling is performed

e	$\overline{ni}$	$\overline{fm}_{<}$	$\overline{fm}_{=}$	$\overline{fm}_{>}$	min qim	max qim	$\overline{qim}$	$\underline{qim}$	$\underline{ci}_{< -0.05}$	$\underline{ci}_{> 0.05}$
frst	0.51	0.23	0.50	0.27	-0.51	0.35	0.00162	0.0031	0.105	0.13
rapdf	0.46	0.22	0.55	0.23	-0.29	0.26	0.00054	0.0012	0.119	0.14
solv	0.51	0.24	0.50	0.26	-0.39	0.35	0.00188	0.0037	0.108	0.14
hydb	0.51	0.31	0.49	0.20	-0.44	0.35	-0.01329	-0.0258	0.281	0.10
tors	0.55	0.27	0.46	0.27	-0.55	0.35	-0.00171	-0.0031	0.128	0.12
SVM	0.40	0.14	0.61	0.25	-0.21	0.29	0.00638	0.0160	0.048	0.16

PVH, all Measures for TM-score, loop modeling is performed

e	$\overline{ni}$	$\overline{fm}_{<}$	$\overline{fm}_{=}$	$\overline{fm}_{>}$	min qim	max qim	$\overline{qim}$	$\underline{qim}$	$\underline{ci}_{< -0.05}$	$\underline{ci}_{> 0.05}$
frst	0.70	0.35	0.31	0.34	-0.43	0.29	0.00047	0.00068	0.109	0.118
rapdf	0.59	0.30	0.42	0.28	-0.35	0.35	-0.00137	-0.00233	0.140	0.126
solv	0.69	0.35	0.32	0.34	-0.42	0.34	-0.00014	-0.00020	0.116	0.133
hydb	0.70	0.46	0.30	0.24	-0.53	0.35	-0.04492	-0.06420	0.428	0.095
tors	0.72	0.39	0.28	0.33	-0.64	0.29	-0.00605	-0.00836	0.152	0.105
SVM	0.58	0.22	0.43	0.35	-0.27	0.35	0.00774	0.01339	0.052	0.150

Table 3: Description of distributions when selecting models according to the FRST potentials and the SVM.
